# Utilization of basic health units of FHS according to private health insurance

**DOI:** 10.11606/S1518-8787.2018052000383

**Published:** 2018-05-07

**Authors:** Leonardo Ferreira Fontenelle, Maria Beatriz Junqueira de Camargo, Andréa Dâmaso Bertoldi, Helen Gonçalves, Ethel Leonor Noia Maciel, Aluísio J D Barros

**Affiliations:** I Universidade Federal de Pelotas. Faculdade de Medicina. Programa de Pós-Graduação em Epidemiologia. Pelotas, RS, Brasil; II Universidade Federal de Pelotas. Faculdade de Odontologia. Departamento de Odontologia Social. Pelotas, RS, Brasil; III Universidade Federal do Espírito Santo. Centro de Ciências da Saúde. Departamento de Enfermagem. Vitória, ES, Brasil

**Keywords:** Health Services Needs and Demand, Health Centers, Health Services, utilization, Supplemental Health, Health Maintenance Organizations, utilization, Equity in the Resource Allocation, Necessidades e Demandas de Serviços de Saúde, Centros de Saúde, Serviços de Saúde, utilização, Saúde Suplementar, Sistemas Pré-Pagos de Saúde, utilização, Equidade na Alocação de Recursos

## Abstract

**OBJECTIVE:**

To describe the utilization of basic health units according to coverage by discount card or private health insurance.

**METHODS:**

Household survey in the area covered by Family Health Strategy in Pelotas, state of Rio Grande do Sul, Brazil, from December 2007 to February 2008, with persons of all age groups. The frequency of (medical or non-medical) healthcare seeking at the basic health units in the last six months and the prevalence of basic health unit utilization for the last medical consultation (in case it had been performed up to six months before, for a non-routine reason) were analyzed by Poisson regression adjusted for the sampling design.

**RESULTS:**

Of the 1,423 persons, 75.6% had no discount card or private health insurance. The average frequency of (medical or non-medical) healthcare seeking was 1.6 times in six months (95%CI 1.3-2.0); this frequency was 55.8% lower (p < 0.001) among privately insured persons compared to those with no discount card or private health insurance. Among the last medical consultations, 35.8% (95%CI 25.4-47.7) had been performed at the basic health units; this prevalence was 36.4% lower (p = 0.003) among persons covered by discount card and 87.7% lower (p = 0.007) among privately insured persons compared to those without both coverages.

**CONCLUSIONS:**

Private health insurance and, to a lesser degree, discount card coverage, are related to lower utilization of basic health units. This can be used to size the population under the accountability of each Family Health Strategy team, to the extent that community health workers are able to differentiate discount card from PHI during family registration.

## RESUMO

**OBJETIVO:**

Descrever a utilização de unidades básicas de saúde conforme a cobertura por cartão de desconto e plano de saúde.

**MÉTODOS:**

Inquérito domiciliar na área de abrangência da Estratégia Saúde da Família de Pelotas, RS, entre dezembro de 2007 e fevereiro de 2008, incluindo pessoas de todas as faixas etárias. A frequência de busca por atendimento (médico ou não) nas unidades básicas de saúde nos últimos seis meses e a prevalência do uso das unidades básicas de saúde para a última consulta médica (caso esta tivesse sido realizada até seis meses atrás, e tivesse tido um motivo que não rotina) foram analisadas por regressão de Poisson ajustada para o delineamento amostral.

**RESULTADOS:**

Das 1.423 pessoas, 75,6% não estavam cobertas por cartão de desconto ou plano de saúde. A frequência média da busca por atendimento (médico ou não) foi de 1,6 vezes em seis meses (IC95% 1,3-2,0); essa frequência foi 55,8% menor (p < 0,001) entre as pessoas cobertas por plano de saúde em comparação às pessoas sem cartão de desconto ou plano de saúde. Dentre as últimas consultas médicas, 35,8% (IC95% 25,4-47,7) tinham sido realizadas nas unidades básicas de saúde; essa prevalência foi 36,4% menor (p = 0,003) entre as pessoas cobertas por cartão de desconto e 87,7% menor (p = 0,007) entre as pessoas cobertas por plano de saúde em comparação às pessoas com ambas as coberturas.

**CONCLUSÕES:**

A cobertura por plano de saúde e, em menor grau, a cobertura por cartão de desconto associam-se a uma menor utilização das unidades básicas de saúde. Isso pode ser utilizado para dimensionar a população sob a responsabilidade de cada equipe de Estratégia Saúde da Família, na medida em que os agentes comunitários de saúde sejam capazes de diferenciar cartão de desconto e plano de saúde durante o cadastramento das famílias.

## INTRODUCTION

Although it is named “supplementary health”^1^, private health insurance (PHI) effectively has a “duplicate” function in Brazil^2^, according to the taxonomy of the Organisation for Economic Co-operation and Development (OECD)^3^. One consequence of this duplicate function is that, to some degree, privately insured persons use services from Brazil's Unified Health System (SUS) even though these services are covered by their health policies. The National Regulatory Agency for Private Health Insurance and Plans (ANS) monitors the utilization of SUS for hospital admissions and high-cost outpatient procedures by privately insured persons. In 2015, it required from PHI operators a refund for approximately 500 thousand admissions and procedures - about one for every 100 privately insured persons^4^.

Surveys find less utilization of primary care of SUS among privately insured people^5^ ^-^ ^12^. However, these estimates might be distorted for not differentiating PHI from discount cards. In a household survey conducted in a medium-sized city (Pelotas, RS), almost half the participants who said to be privately insured were actually covered by a discount card^13^. Unlike PHI, discount cards offer (for low-cost monthly fees) access to a list of health services which are paid through out-of-pocket payments by the patients themselves^13^. Although they offer neither prepayment nor risk sharing, discount cards are considered competitors of PHI^14^ ^,^ ^15^.

The purpose of this article was to describe the utilization of basic health units (BHU) according to discount card or PHI coverage among persons covered by the Family Health Strategy (FHS).

## METHODS

We analyzed data from a household survey conducted in the area covered by the FHS in the urban area of Pelotas from December 2007 to February 2008. The sampling plan was a systematic sampling of households within each microarea (the area under responsibility of a community health worker), proportionally to the number of families enrolled in the FHS. Therefore, we could enroll a household even if it was not registered. All residents of each selected household were invited to be part of the study, except residents unable to participate such as hospitalized, deaf persons with no translators or those with dementia. Residents or, when necessary, their legal guardians answered a questionnaire covering multiple domains: sociodemographic, economic level, health conditions and interaction with public and private health services. The questionnaire was answered preferably by the person (95% of the cases), from the age of 15, and by the father or mother (94% of the cases) among the youngest. Interviewers with complete second degree education, hired and trained specifically for the research conducted the interviews. Further details were described elsewere^13^.

The utilization of BHU was assessed with regard to (medical or non-medical) healthcare seeking and the last medical consultation. Participants reported the number of times they sought any medical or non-medical care at their neighborhood's BHU in the last six months. Participants who sought care at their BHU at least once during this period freely reported the main reason for seeking care (coded *a posteriori*), and assessed care on a five-level scale (from very bad to very good), which was dichotomized in good (very good and good) and regular or bad (regular, bad, very bad).

In cases in which the last medical consultation had been made in the last six months and the reason given was not routine (for example, prenatal, prescription renewal or chronic disease control), participants informed the site of the medical consultation, the main reason for choosing the site (coded *a posteriori*) and their satisfaction with the consultation. Satisfaction regarding the last consultation was evaluated by 10 questions, which were adapted from Kloetzel et al.^16^ by means of a pre-pilot (performed in the area covered by a FHS team, which was not included in the sample of this study^13^) in order to ensure understanding of the questions by the study population. Each of the 10 questions had a five-level response, from very dissatisfied to very satisfied, coded numerically from zero to 10 as described by Kloetzel et al.^16^ Each person's satisfaction was described in the form of a general score, composed by the simple average of the 10 questions.

The main exposure was coverage by discount card or PHI. As described previously^13^, this variable was assessed for each person based on the names of the “health insurance plans” by which the person reported being covered. These names were categorized as discount cards in cases in which health care was paid directly by the person, through direct disbursement. Persons with one or more health insurance policies were categorized as privately insured even if they also had a discount card. Participants for whom it was not possible to verify the nature of the “health insurance plan” could not participate in the analysis.

The covariates of the adjusted analysis were: age (zero to 14, 15 to 24, 25 to 44, 45 to 64, or 65 years or more); sex (male or female); skin color or ethnicity (white/yellow/indigenous and mixed/black); the economic level (assessed by the National Wealth Score^17^ [IEN] and divided into quintiles according to their distribution in the sample) and the self-reported health status, dichotomized in good (very good and good of the original response) and regular or bad (regular, bad, very bad). Skin color or ethnicity was dichotomized because of the small number of indigenous or yellow people, the similarity between them and the white people in relation to the studied outcomes, and for being an adjustment variable whose association with the outcome would not be interpreted. The selection of the covariates assumed sociodemographic variables and the economic level as distal determinants of the utilization of health services and health status as a proximal determinant, along with discount card or PHI coverage.

In the analysis of categorical or dichotomous outcomes, we used the Pearson's chi-square test to test the independence of the variables, and Poisson regression to estimate prevalence ratios. In the case of frequency of healthcare seeking (discrete numerical variable), the Poisson regression was used to estimate the rate of healthcare seeking and to explore the possibility of moderation of the association by socioeconomic and health characteristics. Finally, we used linear regression to estimate differences in satisfaction regarding the medical consultation in the BHU (continuous numerical variable).

Stata software, version 13 (StataCorp. College Station, Texas, EUA) was used to perform the analysis. We used the the *svy* prefix to adjust the analyzes for the design effect, considering the BHU as the primary sampling unit and assigning equal weight to all the observations.

The survey was approved by the Research Ethics Committee of the School of Medicine of the Universidade Federal de Pelotas (Official 133/2006) and consented by the Municipal Health Department, which collaborated in the planning of the survey. Participants were included in the survey after signing a free and informed consent form.

## RESULTS

The survey approached 550 households, with 1,491 residents. After 22 refusals, 13 exclusions due to inability to participate and two contact impossibilities, the survey included 1,454 (97.5%) persons. Thirty-one persons were excluded from this analysis because it was not possible to ensure their coverage by discount card or PHI, remaining 1,423 (95.4%) participants.

Participants had a median age of 32 years, ranging from zero to 95; slightly more than half (52.2%) were female, and two-thirds (66.1%) reported good health status (Table 1). The majority (75.6%) of the participants were not covered by either a discount card or PHI. Discount card or PHI coverage varied with age and socioeconomic level.

**Table 1:** Socioeconomic and health characteristics of the sample according to the discount card or private health insurance coverage. Area of Family Health Strategy coverage in the urban area of Pelotas, state of Rio Grande do Sul, Brazil, 2008.

Feature		No coverage	Discount card	Health insurance	Total
		(75.6%)	(15.8%)	(11.6%)	
Age (years) (n = 1,423)					p < 0.01
	0-14	288	46	30	364
	15-24	145	25	25	195
	25-44	245	41	42	328
	45-64	225	61	43	329
	65 or more	130	52	25	207
Sex (n = 1,423)					p = 0.07
	Male	509	105	66	680
	Female	524	120	99	743
Skin color or ethnicity (n = 1,419)					p = 0.05
	White, yellow or indigenous*	642	164	118	924
	Mixed or black	387	61	47	495
Quintiles of IEN (n = 1,423)					p < 0.01
	1st (poorer)	259	19	10	288
	2°	242	39	7	288
	3°	239	23	25	287
	4°	165	72	47	284
	5th (richer)	128	72	76	276
Self-reported health status (n = 1,423)					p = 0.53
	Regular or bad	352	81	50	483
	Good	681	144	115	940
Total		1,033	225	165	1,423

IEN: National Wealth Score

* 12 yellow skin and 23 indigenous persons.

p values are for testing independence between the respective characteristics and discount card or health insurance coverage.

The mean frequency of (medical or non-medical) healthcare seeking at the neighborhood BHU was 1.6 times in six months (95%CI 1.3-2.0) (Table 2). This frequency was lower among privately insured persons than among those without discount card or PHI (p < 0.01), even after adjustment for socioeconomic and health characteristics (p < 0.01). The association between frequency and coverage by discount card or PHI did not vary according to age group (p = 0.13), sex (p = 0.38), skin color or ethnicity (p = 0.96), IEN (p = 0.39) or self-reported health status (p = 0.41).

**Table 2:** Mean frequency of search for care in the basic health unit of the neighborhood in the last six months according to discount card or private health plan coverage. Area of Family Health Strategy coverage in the urban area of Pelotas, state of Rio Grande do Sul, Brazil, 2008.

Variable		Frequency	Ratio (95%CI)	
		Mean (95%CI)	Crude	Adjusted*
Coverage			p < 0.01	p < 0.01
	No coverage	1.77 (1.40-2.24)	1	1
	Discount card	1.42 (1.07-1.89)	0.80 (0.58-1.11)	0.89 (0.67-1.19)
	Private health insurance	0.78 (0.53-1.14)	0.44 (0.31-0.63)	0.55 (0.42-0.73)
Total		1.60 (1.26-2.02)		

Crude analysis with n = 1,398 and adjusted analysis with n = 1,394.

* Adjusted analysis for age, sex, skin color or ethnicity, IEN quintile and self-reported health status.

Among those seeking healthcare at their neighborhood BHU at least once in the last six months, 61.4% (95%CI 54.1-68.2) evaluated the health care as good (Table 3). This evaluation did not change with discount card or PHI coverage (p = 0.07), even in the adjusted analysis (p = 0.26).

**Table 3:** Evaluation of care at the basic health unit in the last six months according to discount card or health insurance coverage. Area of Family Health Strategy coverage in the urban area of Pelotas, state of Rio Grande do Sul, Brazil, 2008.

Variable		Good care	Ratio (95%CI)	
		% (95%CI)	Crude	Adjusted*
Coverage				
	No coverage	60.7 (54.7-67.4)	1	1
	Discount card	70.5 (59.6-83.5)	1.16 (0.99-1.36)	1.14 (0.94-1.39)
	Private health insurance	50.0 (31.6-79.2)	0.82 (0.55-1.22)	0.85 (0.56-1.29)
Total		61.4 (54.1-68.2)		

Crude analysis with n = 658 and adjusted analysis with n = 655.

* Adjusted analysis for age, sex, skin color or ethnicity, IEN quintile and self-reported health status.

For 80.2% (95%CI 74.8-84.7) of the persons the main reason for seeking health care at the BHU was that it was the closest service. The other most common main reasons were good quality care (7.0%; 95%CI 4.9-9.9) and because they were known in or used to being assisted in the BHU (5.0%; 95%CI 2.7-9.2). The main reason for seeking the BHU did not vary according to discount card or PHI coverage (p = 0.22).

Considering medical consultations in any location, 65.2% (95%CI 61.7-68.6) of the persons had had one or more visits in the last six months. Of these, 73.1% (95%CI 66.0-79.2) had a non-routine reason for the last consultation. Both proportions did not vary according to discount card or PHI coverage (respectively, p = 0.11 and p = 0.18).

Among those persons whose last medical consultation had been in the last six months and had a non-routine reason, 35.8% (95%CI 25.4-47.7) had their last consultation at their neighborhood BHU (Table 4). This proportion was lower both among those covered by a discount card and among the privately insured persons, compared to those without a discount card or PHI (p < 0.01).

**Table 4:** Place of last medical consultation according to discount card or private health insurance coverage. Area of Family Health Strategy coverage in the urban area of Pelotas, state of Rio Grande do Sul, Brazil, 2008.

Variable		Distribution (%, 95%CI)			
		No coverage	Discount card	Health insurance	Total
Place of medical consultation					p < 0.01
	Basic health unit of the neighborhood	43.4 (33.1-54.3)	27.6 (17.8-40.2)	5.3 (1.2-21.0)	35.8 (25.4-47.7)
	Medical office or clinic	7.3 (3.4-14.9)	29.5 (18.7-43.2)	60.0 (43.2-74.7)	18.0 (10.7-28.5)
	Emergency room or emergency care	18.8 (14.8-23.5)	10.5 (4.7-21.5)	13.3 (8.3-20.8)	16.6 (13.4-20.4)
	Others*	30.5 (23.8-38.1)	32.4 (21.3-45.9)	21.3 (16.6-27.0)	29.7 (24.1-35.9)
Total		100	100	100	100

Analysis restricted to persons with any medical consultation in the last six months for a non-routine reason (n = 590).

* Health center outside the neighborhood, hospital, residence, etc. The p value is for the independence test between the variables.

People whose last medical consultation had been held at the BHU had a mean satisfaction score of 8.0 points on a scale from zero to 10 (Table 5). Satisfaction with the last medical consultation at the BHU did not change according to discount card or PHI coverage (p = 0.58), even after adjusting the analysis for socioeconomic and health characteristics (p = 0.55).

**Table 5:** Satisfaction with the medical consultation among persons whose last consultation occurred at the basic health unit of the neighborhood, according to discount card or private health insurance coverage. Area of Family Health Strategy coverage in the urban area of Pelotas, state of Rio Grande do Sul, Brazil, 2008.

Variable		Satisfaction score			
		Median (IQR)	Mean (95%CI)	Crude difference (95%CI)	Adjusted difference* (95%CI)
Coverage				p = 0.58	p = 0.55
	No coverage	8.2 (7.3-9.1)	7.9 (7.7-8.2)	0	0
	Discount card	8.4 (7.8-9.3)	8.3 (7.4-9.1)	0.4 (-0.4-1.1)	0.3 (-0.4-1.0)
	Private health insurance	8.1 (7.6-8.6)	8.1 (7.3-8.8)	0.1 (-0.6-0.8)	0.1 (-0.8-1.0)
Total		8.2 (7.3-9.1)	8.0 (7.7-8.3)		

IQR: interquartile range

Crude analysis with n = 211 and adjusted analysis with n = 210.

* Linear regression model adjusted for age, sex, skin color or ethnicity, IEN quintile and self-reported health status.

For 73.9% (95%CI 62.2-83.0) of the persons, the proximity was the main reason for choosing the BHU as the location for the medical consultation. The other most common main reasons were to know and be known in the service for 8.5% (95%CI 3.9-17.5); impossibility of obtaining the consultation in other service for 5.7% (95%CI 2.9-10.7); and good quality of care for 4.7% (95%CI 2.4-9.3). These reasons did not vary with discount card or PHI coverage (p = 0.05).

## DISCUSSION

This study found variations in the utilization of the BHU according to coverage by PHI and, to a lesser degree, discount card. In an urban population covered by the FHS, the prevalence of utilization of the BHU for the last medical consultation was about 90% lower among privately insured people and about 35% lower among persons covered by discount card, compared to persons without both coverages. In addition, the frequency of (medical or non-medical) healthcare seeking at the BHU was about 55% lower among privately insured persons. The direction of these associations is compatible with previous surveys^5^ ^-^ ^12^, but it is not possible to compare directly their magnitude because the estimates are different.

The reduction in the utilization of the BHU for the last medical consultation was more significant than the reduction of the search for any kind of care at the BHU. One possible explanation is that BHU offer services that are not necessarily offered by PHI or discount cards, such as dental care and medication distribution^18^. Another possible explanation is that even if persons with PHI or discount card used BHU for medical consultations with the same frequency, they would have a lower proportion of consultations performed at the BHU because they used other health services more often than other people^2^ ^,^ ^7^ ^,^ ^19^ ^-^ ^21^. Even though both explanations are probably valid to some degree, this study could not quantify their relative importance due to the lack of information on the number of medical consultations in the last six months inside and outside the BHU.

Another finding of this study was the good evaluation the BHU received by the persons who actually used it. Three-fifths of those who sought care at the BHU evaluated the care as “good” or “very good”, and the overall satisfaction score for the medical consultation was 8.0 on average (on a scale from zero to 10). This overall satisfaction score is close to the general evaluation of the consultation both in the study that originally proposed the instrument^16^ (in a teaching care unit) and in a subsequent study performed on several services in Porto Alegre, state of Rio Grande do Sul^22^ (averaging between services with high and low primary health care orientation score).

This good evaluation of the health care did not vary according to the discount card or PHI coverage. This suggests that access to care in other services does not influence the evaluation of the BHU. However, given the cross-sectional nature of this study, one cannot exclude the possibility of “reverse causality”: that persons covered by discount card or PHI only seek care at the BHU if they do a good evaluation, since it is easier for them to use private healthcare services. In addition, in relation to medical consultation at BHU, it was not possible to accurately estimate the satisfaction of privately insured persons, since only four of them reported having had their last medical consultation there.

A third finding of this study is that the main reason for the utilization of the BHU is the locality, regardless of the coverage or not by discount card or PHI. Other frequently reported reasons, such as the person's relationship with the service (knowing or being known in the service), can be understood as facets of the same reason, since they refer to the characteristics advocated by Brazil's National Primary Care Policy (PNAB) for primary care services^23^. The PNAB establishes accessibility (which includes geographical proximity) and long-term relationship as some of the principles of primary health care and stipulates that the BHU have a defined territory^23^. Therefore, persons usually look for the BHU closest to their home. This helps persons being known by the professionals of their BHU and vice versa.

The limitations of this study need to be considered. As with any survey, reliability and accuracy of estimates depend on the information participants provide. Surveys tend to underestimate the frequency of utilization of health services, both in comparison to administrative data and medical records^24^ and in comparison to journals filled in by the participants^25^. This underestimation increases with extremes of age, frequency of utilization, benignity of the health problem, and the reference period of the question on utilization, among other factors^24^ ^,^ ^25^. Therefore, the utilization and its association with discount card or PHI coverage could have been underestimated. To minimize this limitation, we used a relatively short reference period (six months, instead of 12), and the question regarding utilization was formulated by stating the first month of recollection^24^.

Given the cross-sectional design, establishing with confidence the direction of associations was not possible. While it is reasonable for people covered by PHI or discount card to switch partly from public health services (such as BHU) to private services, it might be that people with a more critical view of public services are more likely to adhere to discount card or PHI. This last possibility is reinforced because in a previous study of this same survey^13^, the main reasons for adhering to a PHI or discount card were “safety” and “quality of care”. In any case, this study aimed not to investigate the reason, but rather to describe the utilization of the BHU by people covered by discount card or PHI.

The validity of the findings is reinforced by the careful planning and execution of the survey. Its sample plan respected the area covered by the BHU, while allowing the inclusion of households that were not registered regardless of the reason. In addition, there was an extensive quality control including double-typing of questionnaires, verification of data consistency and verification of 25% of the interviews (5% in person and 20% via telephone).

Despite this attention, caution is necessary in generalizing the results to populations different from that studied. The participants of this study resided in the area covered by the FHS, in the urban perimeter of a single medium-sized municipality and located in the extreme south of the country. We could not assess how the use of BHU by people covered by discount card or PHI varied with factors such as the model of primary health care, rurality or municipality characteristics. However, household surveys conducted in the urban area of the same municipality showed results comparable to the household surveys of national coverage^26^ ^-^ ^28^. Even if the measures of association are not modified by these characteristics, it is expected that utilization of the BHU by its population vary with the proportion of persons covered by PHI and, to a lesser degree, by discount card.

The results of this study can be applied in primary health care management. While the PNAB establishes that each FHS team be responsible for an average of three thousand persons, that number should vary according to the population vulnerability^23^. As persons with no discount card or PHI require more care by the BHU, the FHS teams can be distributed more equitably, giving more importance to those people. In practice, this application will depend on the quality of information about the proportion of persons covered by discount card or PHI. While privately insured people rarely use the BHU, people covered by discount cards use the BHU similarly to people without both coverages. Usually, the only information available is the proportion of privately insured persons, such as in the Health Information System for Primary Care (SISAB) or in household surveys such as the National Health Survey (PNS) or the Consumer Expenditure Survey (POF). However, in this household survey, some of the persons who declared themselves to be privately insured were effectively covered only by discount card^13^. In the management of primary health care, the proportion of privately insured people will typically be available through SISAB, whose family records are maintained by community health workers. Thus, the actual utilization of the BHU by supposedly privately insured persons will depend on the capacity of the community health workers to differentiate PHI from discount card during families’ registration.

People covered by PHI and (to a lesser extent) discount card use less the BHU, although they evaluate health care in a similar way and report the same reasons for choosing the site of care compared to people without both coverages. This can be used to size the population under the accountability of each BHU or FHS team, to the extent that community health workers are able to confidently establish the difference between discount card and PHI during family registration.
